# Essay Illustrating the Tables of the Medical Statistics of the Tuscan Maremma

**Published:** 1846-01

**Authors:** 


### ART. XVIII.

1. *Saggio Illustrativo le Tavole della Statistica Medica delle Maremme Toscane. Compilata per ordine di S.A.R. il Gran-Duca di Toscana.* Da ANTONIO SALVAGNOLI-MARCHETTI Medico ispettore della provincia di Grosseto.—*Firenze,* 1844.

*Essay illustrating the Tables of the Medical Statistics of the Tuscan Maremma. Compiled by order of H.R.H. the Grand Duke of Tuscany.* By ANTONIO SALVAGNOLI-MARCHETTI, Medical Inspector of the Province of Grosseto.—*Florence,* 1844. Folio pp. 89.

2. *Versuch einer Medicinischen Topographie und Statistik von Berlin.* Von DR. H. WOLLHEIM, praktischem Arzte, Wundarzte und Geburtshelfer. Mit einem Vorworte von DR. J. L. CASPER.—*Berlin,* 1844.

*Medical Topography and Statistics of Berlin.* By DR. H. WOLLHEIM, with a Preface by DR. J. L. CASPER.—*Berlin,* 1844.

WE unite our notice of these works because they belong to one class, not because their subjects correspond.

I. The first work before us is one of a class which we are happy to say has greatly increased both in number and value during the last few years; recording the results of the application of medical science in ameliorating the condition of a people, reforming their habits, and not only remedying their diseases, but pointing out the means of avoiding them; thereby converting a miserable and unhealthy population, into a happy and prosperous community. Perhaps our author has been too intent upon his statement of what the country *is,* to give the necessary attention to the question of what it may *become* under scientific management, but his work still points out the evils to be remedied and their probable causes, which is a great step towards their removal.

The Tuscan Maremma, the principal part of which is the province of Grosseto, comprehends a large extent of territory, partly mountainous, partly hilly, and partly consisting of beautiful and very extensive plains. Its extent is 1,439,999 square *agrarj,* about 1,050,000 of which are hilly or mountainous, 380,000 are plains. Rivers, lakes, and water-courses occupy 54,000, and 344,022 are wooded lands. It lies on the shore of the Mediterranean from San Vincenzio to the river Chiarone, the boundary of the Papal States. Its elevation above the level of the sea varies greatly; part is at the same level, while one mountain is 5298 feet, and the summit of another 3211 above it. The insalubrity of the air is the greatest in the plains, slight among the hills, and altogether imperceptible in the mountains. Its effects are manifested, not only in the plains on the seashore, but also inland, along the courses of the rivers and in their proximity. The effects of the malaria are felt in villages, 714 and 975 feet above the level of the sea, while some towns at the sea level and along the coast are perfectly healthy.

The province of Grosseto lies between 28° 12′ to 29° 6′ longitude, and 42° 22′ to 43° 6′ latitude. The climate varies with the elevation of the district. Twenty-two years' observation have shown, that at sun-rise and mid-day the thermometer is two degrees higher than at Florence in winter, and in summer one degree lower. Scarcely any difference is observed in the barometer. In April and May, the thermometer rises nine degrees in the three hours following sun-rise. The hygrometer exceeds by twenty degrees the limits to which it arrives in Florence, and generally in the evening marks from twenty-five to thirty degrees more than in the morning. Thus the climate of the plains is warm and moist. The scirocco wind frequently prevails, and during the warm season is very injurious. Healthy persons feel themselves depressed, the muscular motions languid, the head heavy and painful, somnolence continual and appetite diminished; convalescents readily suffer relapse, and sick persons become worse. These changes are so constantly observed in the hospitals, that no doubt can be entertained of their cause.

There is a board of health in this province composed of a royal commissioner, an officer of the board of municipal superintendence, and a medical inspector. This board superintends everything relating to the public health, and not only the hospitals, but also the medical men and druggists of the province. There are four large and five small hospitals. The province is divided into sixty-eight circuits, and ninety-three medical men perform the medical duties. There are seventy-two pharmacies, which are divided into two classes at the pleasure of the proprietors, and for each class there is a catalogue of medicines, with which they must be constantly supplied. Every medical man is obliged to return to the board of health, a weekly report of the number of sick entered under his care, and of those who have been cured, died, or have left the district. A similar report is made from the hospitals. The reports furnish the elements of medical statistics, and also give an exact idea of the sanitary condition of each district. Besides the resident practitioners, the government retains seven physicians and one surgeon, to afford their assistance in urgent cases, or during considerable extension of disease.

In 1841, the population was 73,966, of which 36,169 were males, 34,239 females, composing 15,598 families, the ratio being 4·83 to the family. About 12,260 inhabit the plains, and 61,906 the hills and mountains. In the summer 3358 emigrate; but this number varies with the salubrity of the season. In 1841, the legitimate births were 3123, illegitimate 132, marriages 817. The number of inhabitants to the square mile is 42·43 for the whole district. In the most unhealthy districts the proportion is from 12·15 to 18·8. In the most healthy, from 90·61 to 161·79, and at Giglio, a sterile but very healthy rock, 224·87. In the summer, 7539 males and 1433 females came to the plains for agricultural labour.

Before 1840, vaccination was altogether neglected, and in some districts inoculation was still practised with much ill effect. In 1840, laws were carried into effect to guard from inoculation and promote vaccination, and with segregation of every case of smallpox the disease soon ceased. In

1841, 203 persons suffered from smallpox and 16 from varioloid disease; 17 only had been vaccinated, and in 7 only was the cicatrix observed. In 16 of these 17 the disease was varioloid. In two individuals vaccinated during the epidemic of variola, the variola and vaccinia developed themselves contemporaneously, and both pustules went regularly through their course. In these two cases the variola was not confluent. In 1842, 32 cases occurred. Of these, 18 had not been vaccinated; and of the other 16, 11 had varioloid disease, in 4 the cicatrices were spurious, and in 1 no sign of vaccination was discovered. Five only of the 16 had true variola. The belief has obtained in Tuscany, that the vaccine virus by reflected transmission through the human body loses its effect as a preservative, and this belief has been strengthened in proportion with the increasing number of cases of variola developed in persons who had been vaccinated. Accordingly, since 1841, the Tuscan physicians have adopted the plan which has been followed in Lombardy since the occupation of Milan by the French, of vaccinating cows by virus taken from man, and then retaking this virus to serve again for our species. Virus taken in this way from the cow was used with the best result in Grosseto in 2999 persons.

We cannot go through the long table and statistical results given by our author with regard to the diseases of the year from June 1840 to May 1842. The principal points are, that 35,619 cases of illness occurred, and the proportions were intermittent fevers 54·58 per cent., pernicious fevers 1·04, continued gastric fever 8·20, rheumatic fever 4·89, phlegmon 3·03, pleuritis and pneumonia 6·10, remittent fever 4·13, chronic diseases 1·69, chronic diseases of the lungs only 5 decimi per cent. On the whole in every 100 permanent inhabitants of the province, the sick have been in the proportion 35·64, and of 100 temporary inhabitants 30·07. The agriculturalists have suffered in the proportion of 82 per cent. Other classes only 18. Relapses have been 12 per cent. Cures 96·31. Deaths 3·69.

Of 1677 deaths, 249 were from intermittent fever, 236 from gastric and rheumatic, 263 from pleuritis and pneumonia, 64 from dropsy, the result of chronic pleuritis or splenitis. Thus these diseases are the most common and also the most fatal of the province, including about two-thirds of the total deaths. The mean duration of life in the province is 22·50 years, while the proportion of deaths for the whole province is 3·19 for every 100 permanent inhabitants; in the island of Giglio it is only 1·92.

Similar tables follow for the succeeding year when the intermittent fevers were only 39·42 per cent., and the temporary inhabitants suffered rather more than the permanent ones (35·50 to 34·75 of the latter.) In both years the number of widows has far exceeded that of widowers, in the proportion of 4 to 14 in one year, and 2 to 12 in the other. This is explained by the greater exposure of males to causes of disease, and it is stated, that the women of the province commonly marry two or three times. The proportion of male deaths was 61·50, and of female 38·50, in the second year of this report. July, August, September, and October are the months in which fever prevails, very few of those engaged in the harvest escaping the effects of the malaria. The difference of temperature between the day and night in this season is from 10 to 14 degrees, and there is great humidity in the atmosphere. The inhabitants of the neighbouring mountains descend in parties of fifteen or twenty for harvest labour, bringing women with them, and even before their arrival they commence the abuse of wine, spirits, and venery. When in the plain they frequently sleep in the open air, or more commonly in large open huts, women and men together. Their diet consists in the morning of bread, often not good, and cheese ; at mid-day, of bread boiled into a sort of soup with water, seasoned with onions, oil, and vinegar, and eaten with the hands ! and in the evening, of what they call *acqua cotta*, which is bread, boiled as before, and seasoned with salt, oil, and pepper. Their drink is wine, often spoiled or made more spirituous with brandy, and water, which is generally of a bad quality. Their labour is most fatiguing, because they work by the piece and not by the hour, and in fields where there is no shade from the concentrated rays of the sun. In order to escape the heat as much as possible, they work very early in the morning, and so become exposed to a dew, so heavy as completely to wet their clothes. The labourers on the ditches and embankments, and those employed in the manufacture of charcoal and potash, work under the same circumstances as the agricultural labourers, and suffer in the same proportion, and the military also from exposure when on sentry duty. Twenty-four cases of malignant pustule occurred, in every case from the patient having touched or eaten flesh of animals dead from a similar disease. It is stated that in some cases, no local contact took place, and that the disease was developed solely from the sufferers having eaten the diseased flesh.

When speaking of the *cause* of the fever, the author inclines to the belief that the mixture of salt with fresh water in the marshes greatly increases the intensity of the miasmata, because pestiferous marshes have become almost innocuous as soon as the ingress of salt water has been prevented. He gives instances of this, one in particular where near Viareggio, a pleasant town has sprung up, now selected as a watering-place, in the very months when before the district was uninhabitable. The phenomena resulting from the admixture of salt and fresh water are also developed, when the bottom of a pond or lake is composed of marine mud, that is to say, when it has formed part of the bed of the sea ; and also when the old bed of the sea, covered by a thin stratum of earth, becomes wet with rain water. Miasmata are evolved by the decomposition of the marine elements, acted upon by the filtration of fresh water, and easily rise into the air by holes and fissures in the stratum of earth produced by acidity. Many parts of the maremma are of this formation.

The *Salmastraie* is supposed also to cause the evolution of miasmata. This term implies the extent of soil covered by saline efflorescence, formed of a thin stratum of earth, which makes a sort of natural filling up (colmata naturale) to an old marine marsh. This marshy bottom, called *cuora marina*, is composed of animal and vegetable substances more or less decomposed, and of hydrochlorate and carbonate of lime and soda. If the superimposed earthy stratum be very thin, it offers but a slight obstacle to the miasmata formed below by chemical decomposition. When it is of such a thickness as to prevent their free escape, a saline efflorescence of the hydrochlorates and carbonates appears in a given point, extends itself, and at last acquires such an extent as to cover several square miles. When this efflorescence appears, the pre-existing vegetation becomes languid, soon dies, and disappears altogether, while gradually another vegetation adapted to the saline nature of the soil shows itself.

The putrefaction developed in marine animal and vegetable bodies, by means of fresh water, and vice versâ, undoubtedly gives rise to emanations extremely injurious to the human organism, in the opinion of our author; and he says, the idea has been proved to be correct by the experiments of Savi upon the putrefaction of *Cara,* and from repeated observations upon the putrefaction of the *Aliga,* when on the sea-shore it is bathed with fresh water.

In some cases, the mixture of salt water in the marshes does not arise from the entrance of sea-water, but from mineral springs which contain sea salt.

The author insists strongly on the influence of the scirocco wind, either in rendering the emanations more deleterious, or in depressing the human organism so as to render it more susceptible to their effects. He compares these effects with those resulting from the admixture of salt and fresh water, the scirocco wind which is impregnated with saline particles, causing an admixture of sea and land air.

Dr. Salvagnoli's remarks on the influence of wooded land upon the health of a district are so sensible, that we extract them, not only as a good specimen of his style, but also as they may afford more useful hints to such of our readers as are interested in the study of medical topography.

"The presence of crowded and extended woods, according to some, and on the contrary their destruction, according to others, cause malaria. Targioni, on the authority of Doni, considered woods injurious, not only for being liable to retain and imprison the principle constituting malaria, but also from being, as he believed, capable of producing them. Brocchi, on the contrary, asserts that in Rome the air was deteriorated by cutting down the woods. Thouvenet, Virey, and Gioia also record facts in favour of the supposition of the utility of woods. Such a disparity of opinion proves, in my judgment, that there are circumstances in which a too extensive and general clearing of woods may be equally injurious, as allowing trees and shrubs to increase and multiply without the regulation of man. We find certain districts and houses, with a perfectly healthy atmosphere, in the midst of extended woods, while others in similar situations, suffer from malaria. It appears to me reasonable to say, with Savi, that woods are injurious only accidentally, and never by themselves. Indeed, it is known that the watery vapours evolved into the atmosphere by woody plants, are in less quantity than those which they absorb; that instead of pouring into the air principles injurious to life, they emit a most useful one, oxygen, and remove hurtful ones, as carbonic acid. It is said against forests, that where they cover the soil drainage is more difficult, and therefore that swamps frequently follow. This is true only with regard to woods in valleys and plains, but even then it is not the woods which produce malaria, but the stagnant waters contained in them; and then of this evil the men must blame themselves who allow the former to increase near their habitations, and do not open or maintain the necessary drains. It is said, lastly, that woods prevent the free action of the north wind; this is a defect inherent only to their situation respectively to towns and districts, and to lakes and marshes that need to be purified by north winds. Woods are useful with respect to the salubrity of the air,—1, because they absorb much humidity from the soil, and render it drier and more healthy;—2, because all plants, and particularly those with falling leaves, remove from the air principles unfavorable to respiration, and distribute a large quantity of oxygen;—3, because they defend the soil from the too ardent rays of the sun, and too great intensity of frost, and thereby prevent the alterations and decompositions which frequently cause injurious effluvia;—4, because they help to impede the direct action of the Scirocco and Lebeccio winds, the former injurious to the health of the inhabitants, the latter to the fertility of the country." (pp. 44-5.)

It appears evident, that the specific cause of the fever of this Maremma is some principle evolved during the decomposition of animal substances in the marshes, and the humidity of the atmosphere and changes of temperature, however great, can only be regarded as auxiliaries, as they exist in as great a degree in districts not subject to diseases of an intermittent type. It is still curious to observe the relations between the changes of season, and the type of the diseases of the Maremma. From January to June, the diseases peculiar to temperate regions are observed, and during the other months of the year in proportion with the increase of atmospheric temperature, fevers become aggravated according to their original force, and pass gradually from intermittence to remittence, and from this to continuity, and assume the aspect of diseases peculiar to southern regions. These phenomena may be expressed by the following formula: in direct ratio with the simultaneous increase of heat and of atmospheric humidity, owing to the influence of climate, miasmatic diseases pass from intermittence to continuity, and become at the same time always more severe.

The period of latency or incubation of the miasma after its introduction has not been determined.

A full account is given of the symptoms of the fever and of the post-mortem appearances, but we find nothing peculiar to the district in question. The most frequent type is the quotidian; the tertian, so common in the north of Europe, is not unfrequent in the winter in the Maremma. When the quotidian fevers of summer prolong themselves into the winter they readily pass to the tertian and quartan type. The numbers in three years were remittents 2066; quotidian 39,923; tertian 10,192; quartan 1544; pernicious 1346.

The access of these fevers in nine cases of ten, occurs during the day; in quotidian, generally in the morning and at mid-day; in tertian and quartan, in the afternoon and towards evening.

We now come to a highly interesting question which has lately excited considerable attention, and upon which the researches of our author are calculated to throw some little light,—the antagonism between the causes of intermittent fevers, and those of phthisis pulmonalis.

From a table showing the diseases of the permanent inhabitants of the Maremma for three years, it appears, that of 81,731 sick, there were only 100 cases of phthisis, or one phthisical patient to every 817. According to Broussais, in Algiers where intermittents prevail, of 40,000 sick in the French army only 62 were phthisical, that is, 1 to every 650 sick, and 1 death to 102 deaths from other causes; while in France there is 1 death to 5 from other causes, and in England the mortality from phthisis, constitutes a sixth part of the whole mortality. Scrofula is also very rare in the Maremma, and also in Algiers; but the author does not explain what he means by this term, and as he states, that three fourths of the phthisical have been previously scrofulous, we conclude that, probably his distinction between scrofula and phthisis, is more an imaginary than a pathological one.

Our author overlooks one important circumstance, the due consideration of which tends to vitiate the whole of his conclusions; it is this: he, as indeed almost all who have treated of this matter, seems entirely to forget the fact, that their observations can only apply to *overt* or confirmed disease, *post-mortem* examinations not being *general;* whereas, all that is essential of the malady (tubercles) may have existed in those dying of fever, without being detected. Moreover, where so many are carried off in early life by another disease (fever) we may reasonably infer, that a certain proportion of them might have become phthisical had they lived. We might as reasonably conclude, that phthisis was a most rare disease among the soldiers of Napoleon, if we comprehended in our calculations the hundreds of thousands who fell under the sword of the enemy, or the inclemencies of climate.

Interesting physical and chemical observations on the blood of the inhabitants of the Maremma have shown, that the properties of blood, taken even in simple inflammations, differ greatly from those of the blood of inhabitants of healthy districts.

"So great and so constant is the difference, that from the physical examination of the blood only, almost without error, the physician may judge if the patient resides constantly in an unhealthy atmosphere, and if the liver and spleen have become altered." (p. 65.)

"The blood is very black, forms a coagulum, which I could scarcely call grumous, by no means tenacious, separating, when first drawn, a very little serum, and which breaks up without any resistance to a round blunt stick; when the stick is passed through the coagulum, it does not present sufficient resistance to be raised: occasionally it is covered by a stratum, more or less thick, of a yellowish substance, of gelatinous consistence: it never forms the true tenacious, resisting, concave coat, and never or almost never that compact, contracted, conical coagulum, separating a large quantity of serum, by which the phlogistic diatheses are principally signalized. The coagulum readily breaks up or dissolves, (si squaglia,) and then separates a large quantity of yellow, opaque, or greenish serum. These physical qualities of the blood are most marked in the summer and autumn. A more tenacious coat, and more resisting coagulum, are more common in the winter, and especially when the north wind prevails." (pp. 65-6.)

Chemical analysis has shown a regular diminution in the quantity of fibrin, albumen, and fat. Cholesterin which ought not to be found separated from the other constituents of the bile was found in appreciable quantity. We have altered the table of M. Salvagnoli, giving only the parts in 1000, of the fibrin, &c.; he has given their actual weights, which is here unnecessary. The blood examined was taken from four individuals.

No. 1. Had suffered eighteen months from intermittent, and had engorgement of liver and spleen.

No. 2. Had inhabited the district twelve years; was suffering from quartan; liver and spleen also enlarged, the former very painful.

No. 3. Inhabitant of five years' standing; suffered from intermittent for seven months; slight engorgement of liver; extraordinary enlargement of spleen.

No. 4. Inhabitant of nine years; suffering from tonsillitis; had undergone long courses of endemic fevers; spleen engorged but indolent.

| | Fibrine. | Fat. | Colouring Matter. | Albumen. | Water and Salts. | REMARKS. |
|---|---|---|---|---|---|---|
| | Parts in 1000. | | | | | Salts and other substances. |
| 1 | 2·20 | 0·15 | 211·27 | 48·71 | 737·67 | Chlorides, lactates, almost absolute deficiency of phosphates, cholesterin. |
| 2 | 2·06 | 0·21 | 235·63 | 56·61 | 705·49 | Chlorides, lactates, almost absolute deficiency of phosphates, much cholesterin, biliary colouring matter. |
| 3 | 2·29 | 0·13 | 217·54 | 47·59 | 732·45 | Chlorides, lactates, almost absolute deficiency of phosphates, smaller quantity of cholesterin than in the former cases. |
| 4 | 1·96 | 0·16 | 135·61 | 53·10 | 809·17 | Chlorides, lactates, phosphates, and an appreciable quantity of the colouring matter of the bile without a trace of cholesterin. |

The remarks on *treatment* are sound, but do not add to our previous knowledge. Some experiments, however, were made to determine the relative value of the different salts of quinine, and of various other substances. We regret that the number of these was not more limited, as those employed would then have been tried upon a greater number of patients, and the results consequently much more worthy of confidence. Twelve preparations of quinine were employed, and on the whole the sulphate found most beneficial, but when given as usual dissolved in water with excess of sulphuric acid, gastric and cephalic symptoms were very common, and often rendered a discontinuance of the medicine necessary. This was obviated by manufacturing a crystallized neutral sulphate of quinine, which is soluble in eleven times its volume of distilled water. This hint is worth the notice of our pharmacologists.

Of the other substances experimented upon, although none have shown an antiperiodic action equal to that of quinine, still the camomile, the extract of fillirea, (Fillirea latifolia), fillirina and its sulphate, the extract of olive, and a substance called olivina, the bitter principle of the olive leaves, are worthy of considerable attention. The results of their exhibition were very satisfactory in many bad cases during the worst part of the season. On economical grounds alone, a trial of these remedies in periodic diseases should be made in our hospitals. Olive leaves, and the fillirea might be readily procured at a very trifling expense, and perhaps render us great assistance in case of any deficiency in the supply of cinchona. The powdered camomile flowers succeeded well, but it was difficult to give a sufficiently large dose. Might not the active principle be prepared for the market?

Salicine and arsenious acid were both employed without good effect. The fillirea and fillirina were used in seventy-two cases. The camomile powder in twenty. Salicine in four. Arsenic in sixteen. Olivina and the extract of olive in fourteen. Preparation of quinine in seventy-seven.

We have thus gone through the valuable work of M. Salvagnoli, and have condensed into this article such of its contents as we believed likely to be useful or interesting to English practitioners. The style is clear and simple, the reasoning generally sound, and our only regret is, that the statistical information was not presented in a more condensed form, and for a longer period, and that M. Salvagnoli had not devoted a chapter to the means of remedying the evils he has so well described.

II.—The second book on our list is one of those minute and laborious works in which a German author delights, but which an English reviewer approaches with a feeling akin to despair. The multiplicity of subjects of which it treats, and the very slender tie by which they are held together, render a connected account of it next to impossible. All that he can hope to do, is to select a chapter here and there, and cull from it such facts as promise the largest share of amusement and instruction. This is our aim in the present short notice; we shall, therefore, clear at a leap the first fifty pages, with the commendatory preface of Dr. Casper, and alight on the chapter treating of the population of Berlin.

In the interval from 1590 to 1841, the population of the city increased from 12,000 to 321,505. This increase was far from regular, for in the year 1661 the number of inhabitants fell as low as 6500. The population for the year 1841 presents a remarkable approach to equality between the number of male and female inhabitants, the former being 160,802, and the latter 160,703. To the males, however, will have to be added the military, to the number of 11,744. This approach to equality in the number of male and female inhabitants is only observed in the one year 1841, the females exceeding the males in all the preceding years, with the exception of 1840, when the males were slightly in excess. The 321,505 inhabitants, male and female, were thus distributed according to age. Up to the end of the fifth year, 38,815; from the end of the fifth to the end of the seventh years, 12,286; seven to fourteen, 38,162; fourteen to sixteen, 10,803; sixteen to forty-five, 183,904; forty-five to sixty, 31,673; sixty and upwards, 15,862. The males exceed the females up to forty-five years of age, but fall considerably short of them after that period.

From 1820 to 1840 the civil inhabitants of the city increased by 105,756, of which increase, 24,626 was the excess of births over deaths, and 81,130 the excess of immigrants over emigrants.

The number of married persons in Berlin, in the year 1841, was 42,752 males and 42,864 females, the excess of females being accounted for by the circumstance of several of the husbands being engaged in travelling or being in prison beyond the limits of the city. The number of births for the same year was 10,757, which gives rather less than one birth annually to four married couples. The average number of children to a marriage was $3\frac{1}{8}$, or, after some necessary correction, 3.

The proportion of births to the total population, on an average of years, is 1 to 27, and, for the year 1841, 1 to 30-31. The male births were to the female as $1\frac{1}{18}$ to 1, in other words, out of 37 new-born infants, 19 were males, and 18 females. There was one marriage to 100 inhabitants.

Twin births formed $\frac{1}{106}$ of the total births, and there was one triplet in 6279 births.

Such are the principal contents of the chapter on population, from which, passing over some eighty pages treating of matters of no medical interest, we turn to the chapter on the morality of Berlin, which opens with a passage, in justification of our choice, setting forth the intimate relation which exists between morality and health. The first point to which our attention is drawn, is the proportion of illegitimate births. It appears that, on an average of seven years, there was one illegitimate birth to $6\frac{9}{10}$ legitimate, or $14\frac{1}{2}$ per cent. Among the Roman Catholic inhabitants, we are told, the proportion is not much better, but among the Jews it is nearly six times better than the general average. This proportion of 1 to 9 contrasts favorably with several of the large continental cities. In Jena it is 1 to 7; in Stockholm, Dessau, and Göttingen, 1 to 6; in Dresden and Leipzig, 1 to 5; and in Paris, 1 to $3\frac{1}{2}$. St. Petersburg presents the very favorable proportion of 1 to 20. The statistics of suicide present some points of interest. In the interval from 1788 to 1797, only six suicides a year occurred in the city of Berlin; from 1799 to 1808, twelve a year; but from 1813 to 1822, as many as fifty-four. This increase is out of all proportion to the increase of population, for during the first period, the proportion which suicides bore to the population was 1 to 26,000; during the second period 1 to 13,000; and during the last period, 1 to 3000. The proportion of female to male suicides at these three periods respectively, was 1 to 8, 1 to $3\frac{1}{2}$, and 1 to $5\frac{5}{17}$. The crime was most frequent between the ages of 40 and 50, least so between 70 and 80; but one case is recorded under 10 years, and two above 80. The most common causes were insanity and drunkenness; fear of punishment and want ranked next; and religious enthusiasm was the least common cause. The greater number of the criminals belonged to the class of artizans. The favorite mode of death in Berlin was by hanging; next to this by the pistol. In Paris, where suicide, like everything else, must be done with *eclat*, the pistol or a leap from a window is preferred. To the acknowledged suicides must be added a certain proportion of 70 bodies found in the water in the course of two years. The season of the year does not appear to have exercised the same influence on the frequency of suicide as it has been shown to have done at Paris.

The number of suicides at Berlin, when compared with that occurring in several large cities, is considerable. During the last-named period (1813 to 1822) it was, as we have seen, 1 in 3000. In Naples it was 1 in 27,230; in London, 1 in 21,491; in Milan, 1 in 18,021; in New York, 1 in 9474; in Hamburgh, 1 in 4500; in Leipzig, 1 in 3143; in Paris, 1 in 2215; and in St. Petersburg, 1 in 416 (?) This chapter on the morality of Berlin concludes with the somewhat startling statement, that a separation takes place for every 200 or 300 marriages, a fact which points either to a low state of morals or to unusual facility of divorce.

We now pass on to the chapter on the mortality and duration of life in Berlin. In the interval from 1819 to 1841 there died on an average 1 in 36 of the inhabitants. The numbers dying, at different ages, were as follows. Out of 1000 living of both sexes, the deaths during the first year amounted to 282 males and 266 females; under 10 years the numbers were 465 males, and 473 females; under 20, 492 males and 502 females; under 30, 578 males and 565 females; under 40, 650 males and 633 females; under 50, 734 males and 706 females; under 60, 822 males and 783 females; under 70, 907 males and 870 females; under 80, 971 males and 957 females; under 90, 999 males and 995 females; and under 97, 1000 of both sexes. The living for the same periods are easily ascertained by a simple process of subtraction. Still-births were in the proportion of $\frac{1}{18}$ of the total mortality.

Several tables of considerable interest to the statistician will be found from page 353 to page 386, but our limits will not allow us to do more than refer to them for information.

We have noticed only a few of the contents of this very laboriously compiled volume. Those who wish for very minute and trustworthy information on almost any imaginable subject which can be comprised under the two heads of medical topography and statistics, are referred to Dr. H. Wollheim's work, where they will most probably find what they are in search of.

---

